# Supplementary figures and images for: Nuclear dengue virus NS5 antagonizes expression of PAF1-dependent immune response genes
Source: PLoS Pathog. 2021 Nov 19;17(11):e1010100. doi: 10.1371/journal.ppat.1010100 (PMC8641875; doi:10.1371/journal.ppat.1010100)

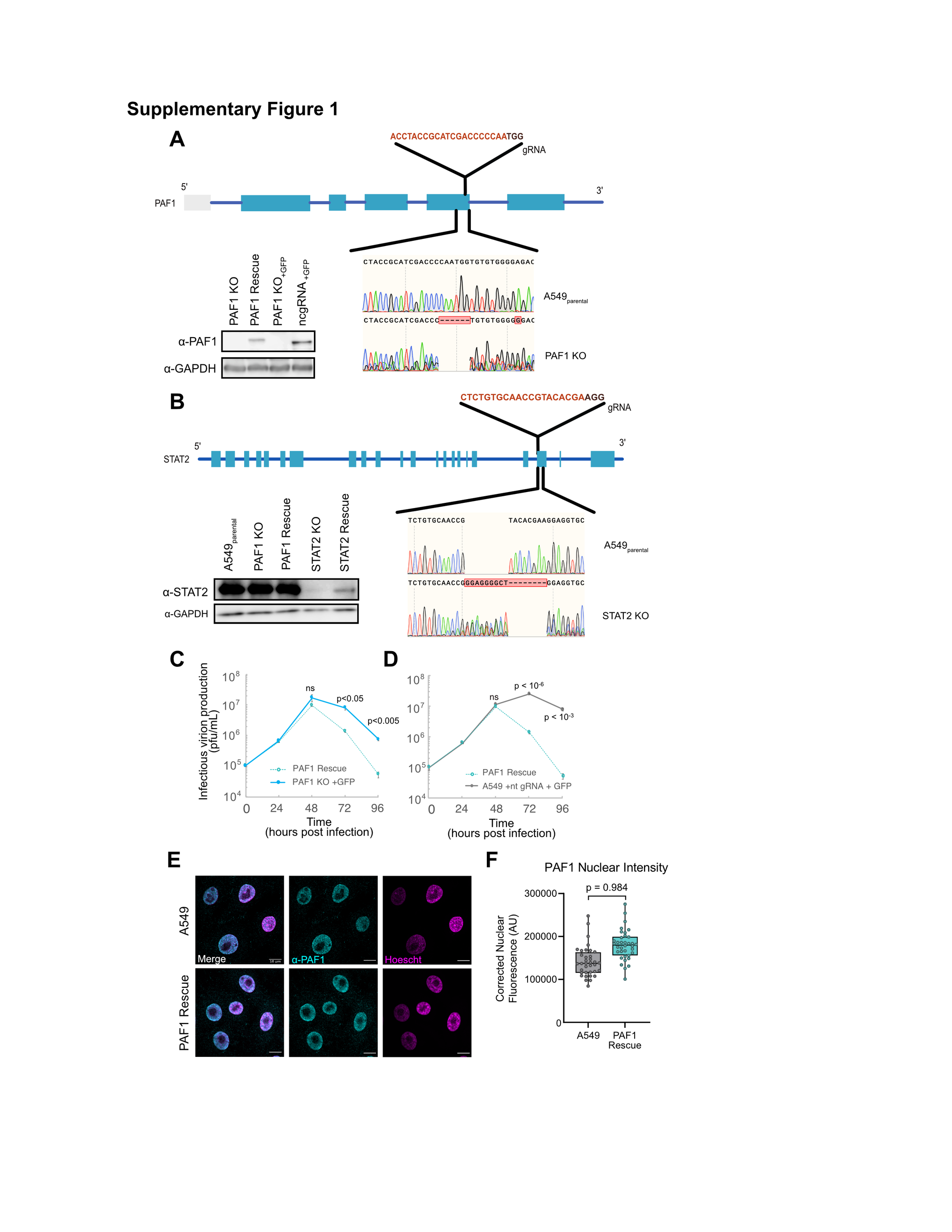

Supplement: S1 Fig — (A) PAF1 gRNA target region and Sanger sequencing of the CRISPR/Cas9 induced deletion. Immunoblotting was performed on protein extracted from parental A549, PAF1 KO and PAF1 rescue. Immunostaining with PAF1 antibody showed the protein depletion for the PAF1 KO cells. PAF1 rescue data showed the restoration of the PAF1 detection. GAPDH is the control for protein loading. PAF1 gRNA target region and Sanger sequencing of the CRISPR/Cas9 induced deletion. (B) STAT2 gRNA target region and Sanger sequencing of the CRISPR/Cas9 induced deletion. Immunoblotting was performed on protein extracted from parental A549, PAF1 KO/rescue, and STAT2 KO/rescue. Immunostaining with STAT2 antibody showed the protein depletion for the STAT2 KO cells. STAT2 rescue data showed the restoration of the STAT2 detection. GAPDH is the control for protein loading. DENV2 replication in (C) PAF1 KO expressing GFP and PAF1 rescue cells and (D) PAF1 rescue and A549 + nt gRNA (non-targeting gRNA) + GFP, MOI 0.1. Data from three replicates are plotted as mean values +/- standard deviation. P values were calculated using a paired, one-tailed Student’s t-test. (E) Heterogeneity of PAF1 expression (cyan) was determined by immunostaining and confocal microscopy. Nuclei were stained with Hoechst (magenta). Scale bar represents 10 μm. A representative image is shown. (F) Quantification of PAF1 nuclear signal intensity across 36 nuclei. P-value was calculated using a F-test. Abbreviations: plaque forming units (pfu), not statistically significant (ns). (TIF) [file ppat.1010100.s001.tif]

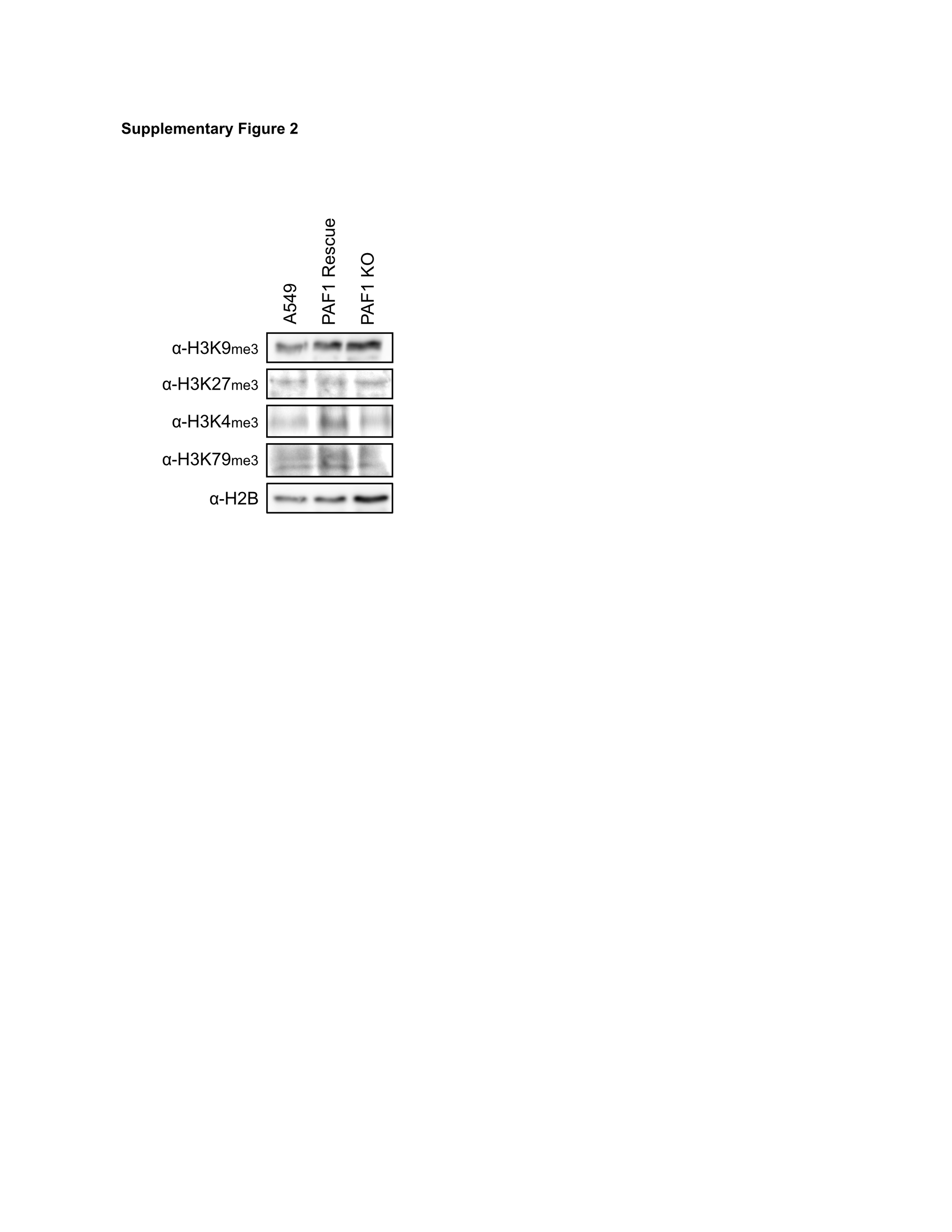

Supplement: S2 Fig — Comparison of global methylation levels in parental A549, PAF1 KO and PAF1 rescue cells. Immunoblotting was performed on protein extracted from parental A549, PAF1 KO/rescue. Immunostaining with H3K9me3, H3K27me3, H3K4me3, H3K79me3 and H2B antibodies showed unchanged level of detection across the different cell lines. (TIF) [file ppat.1010100.s002.tif]

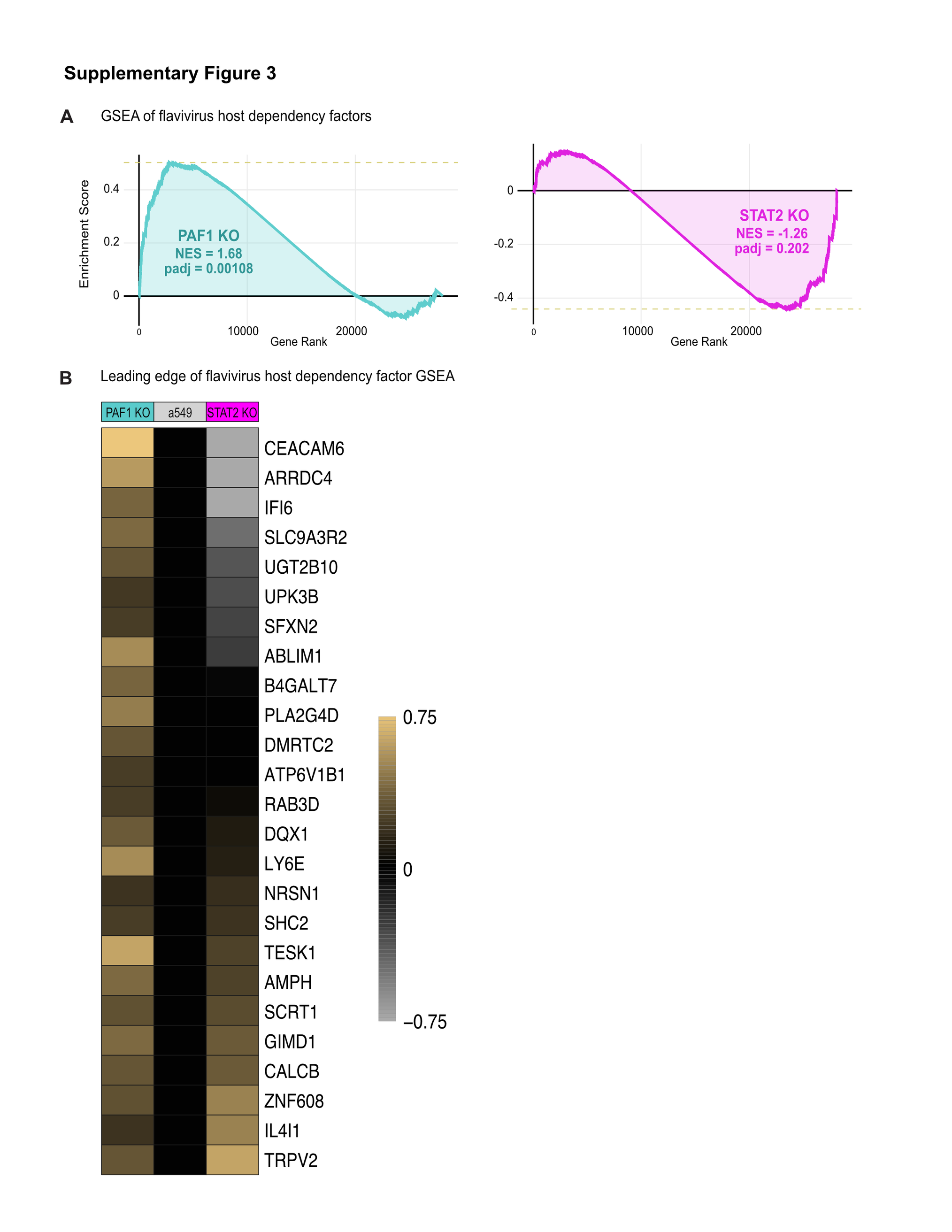

Supplement: S3 Fig — (A) GSEA was performed using list of flavivirus host dependency genes. (B) Leading edge of flavivirus host dependency factors from GSEA. Heatmap represents log2 fold change relative to parental A549 following poly(I:C) treatment. (TIF) [file ppat.1010100.s003.tif]

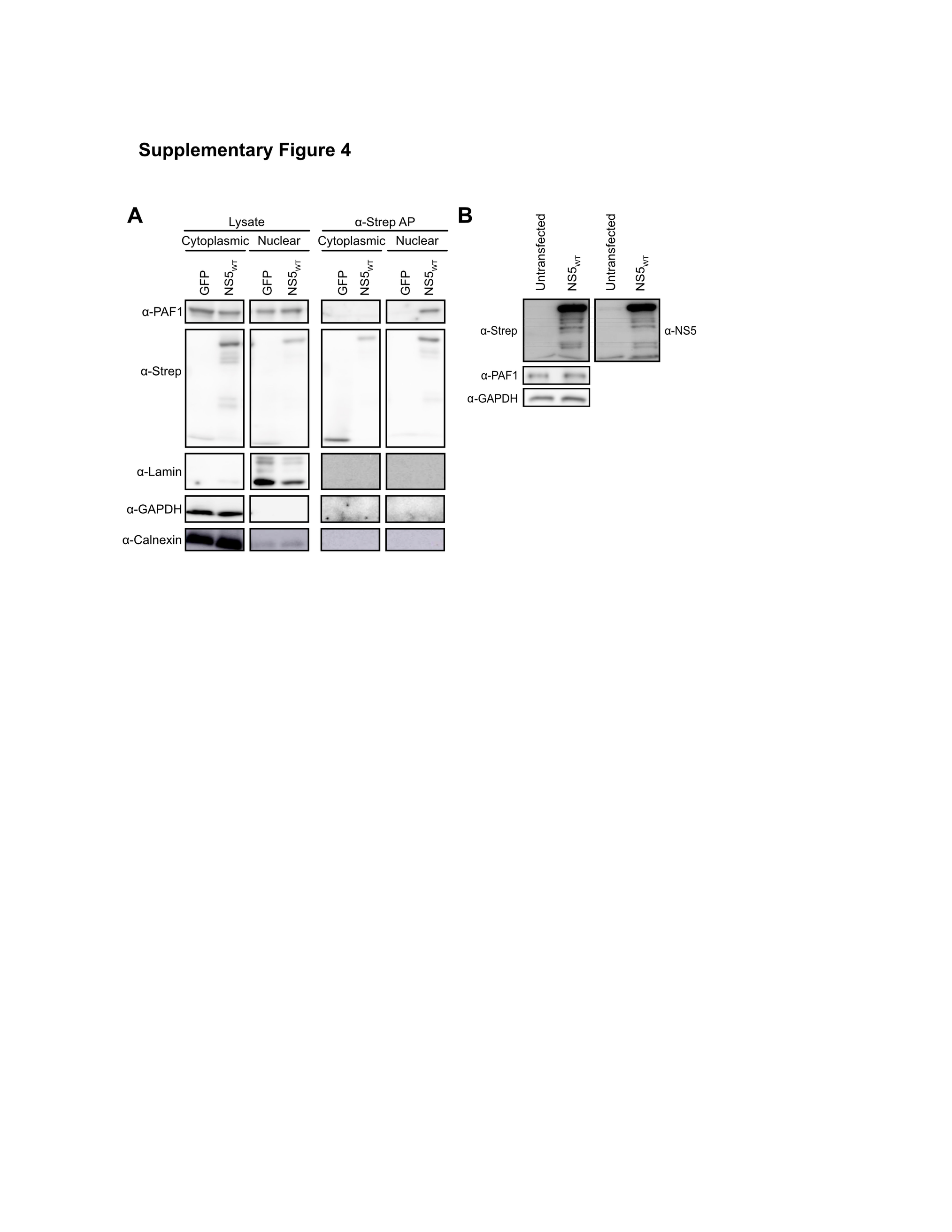

Supplement: S4 Fig — (A) Following nuclear/cytoplasmic fractionation, NS5 and GFP were subjected to affinity purification and immunoblot. Lamin and Calnexin served as controls for fraction purity, GAPDH is the control for protein loading. (B) Immunoblot were performed on parental A549 cells transfected with 2xStrep II tagged DENV2 NS5. Immunoblot was probed with Strep, NS5 and PAF1 antibody. Similar band pattern is observed for NS5 and Strep staining. GAPDH is the control for protein loading. (TIF) [file ppat.1010100.s004.tif]

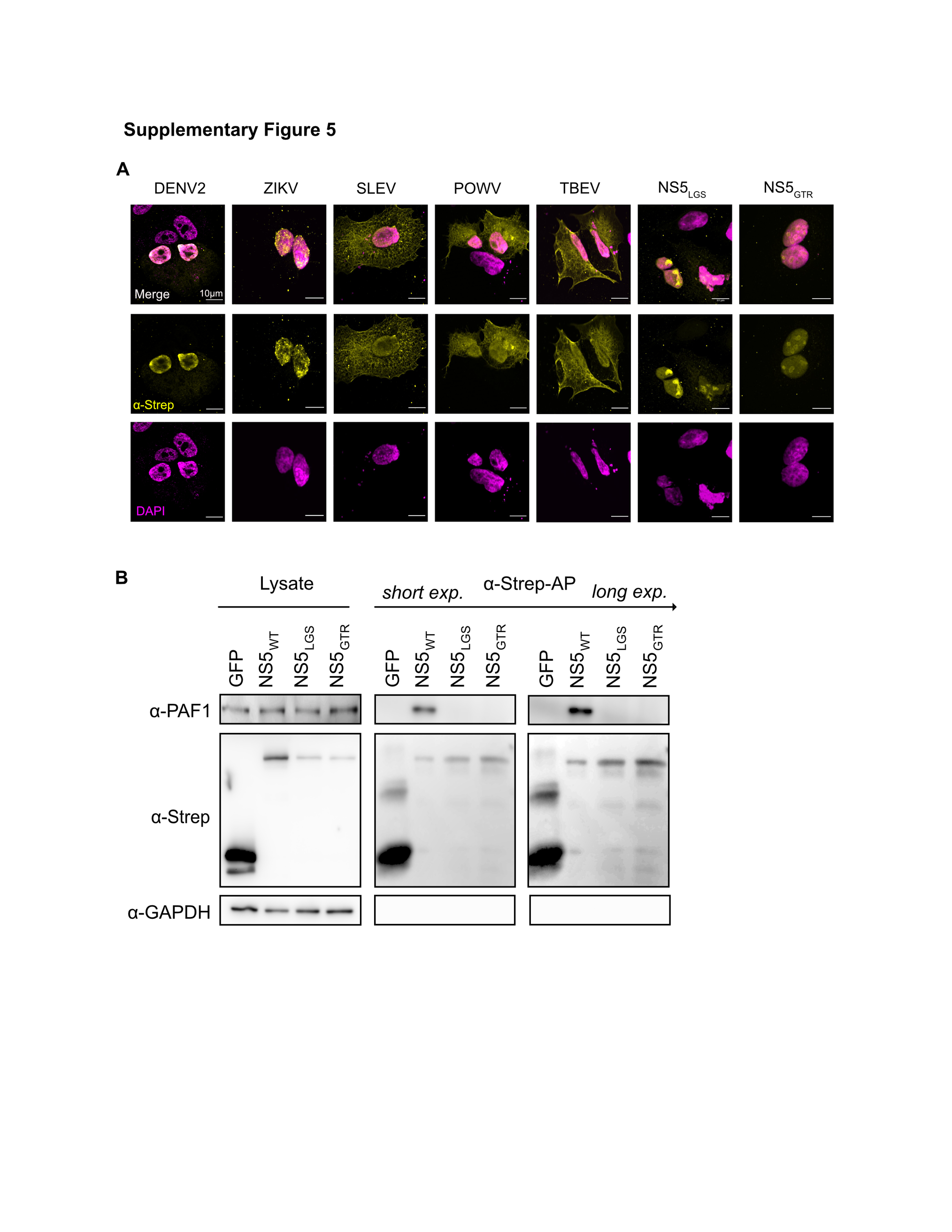

Supplement: S5 Fig — (A) Subcellular localization of 2xStrep II tagged flavivirus NS5s, NS5LGS and NS5GTR (yellow) was determined by immunostaining and confocal microscopy. Nuclei were stained with Hoechst (magenta). Scale bar represents 10 μm. (B) 2xStrep II tagged NS5s (NS5WT, NS5LGS and NS5GTR) were tested for an interaction with PAF1C biochemically. Affinity purification and immunoblot analysis were conducted on protein extraction from parental A549 cells transfected with NS5WT, NS5LGS or NS5GTR. PAF1 antibody was used to identify the PAF1-NS5 interaction. Only NS5WT showed a band for PAF1 staining, at both short and long exposure (x5). GAPDH is the control for protein loading. (TIF) [file ppat.1010100.s005.tif]

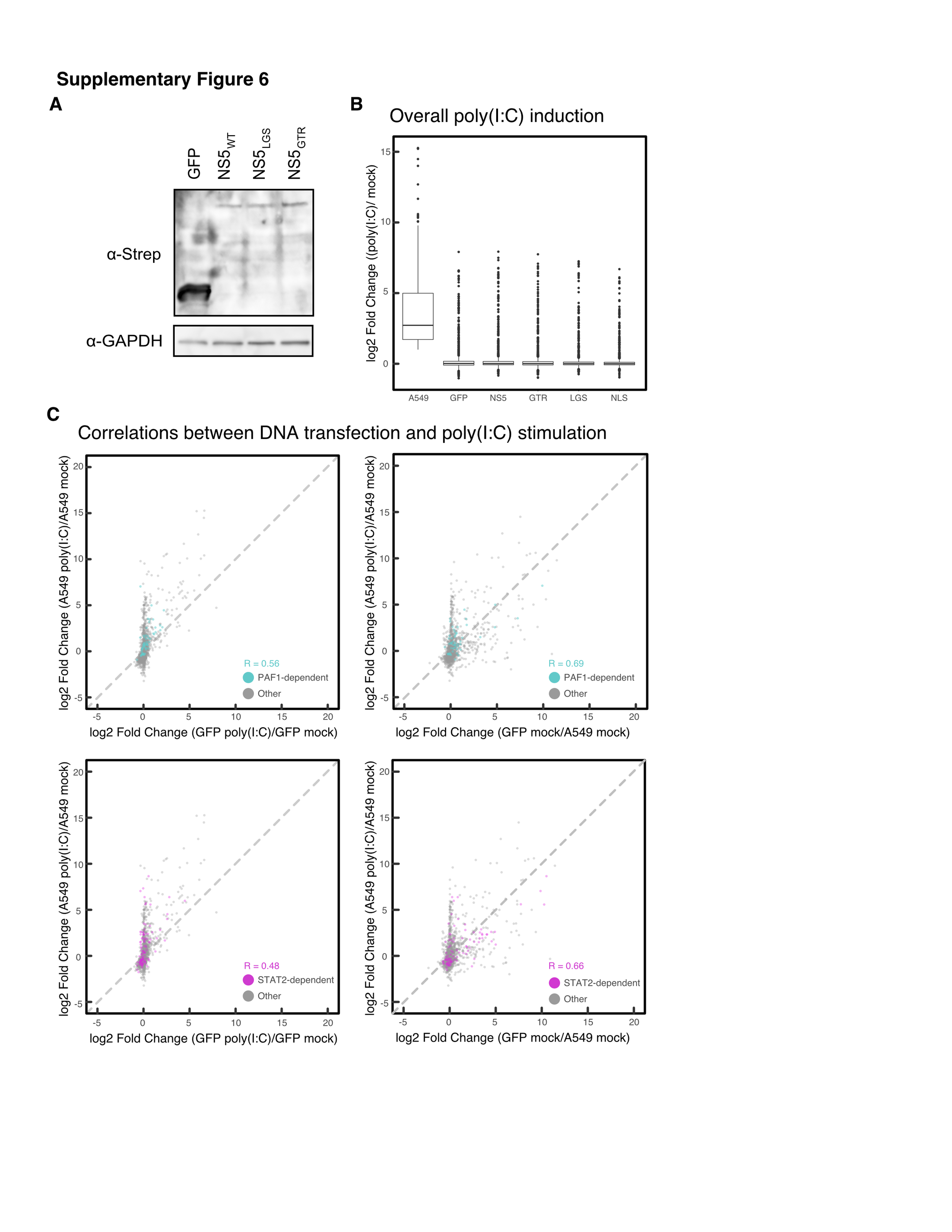

Supplement: S6 Fig — (A) Comparison of NS5 expression in parental A549 transfected with NS5WT, NS5LGS, NS5GTR and NS52xNLS. Immunoblotting was performed on protein extracted from transfected parental A549. Immunostaining with Strep antibody detected an equal level of transfected NS5s for all constructs. GAPDH is a control for protein loading. (B) Changes in gene expression caused by poly(I:C) treatment are shown for the subset of immune response genes (GO:0006955) significantly upregulated for poly(I:C)-treated parental A549 cells relative to mock-treated A549 cells (log2 fold change > 0.5, padj < 0.05). (C) Pearson’s correlation coefficients were calculated for differential gene expression comparing DNA transfection and poly(I:C) stimulation. (TIF) [file ppat.1010100.s006.tif]

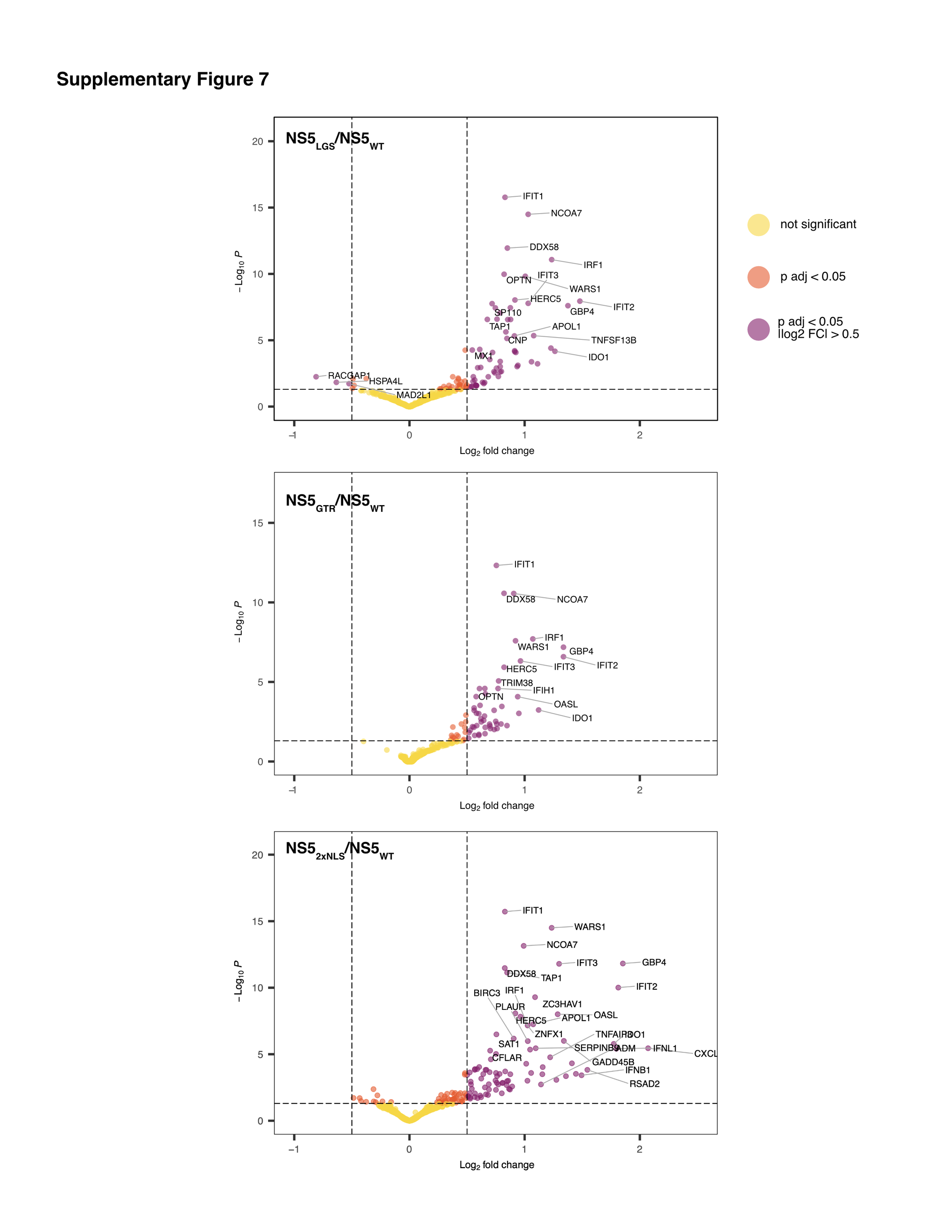

Supplement: S7 Fig — Relative change in gene expression was plotted as log2 fold change versus adjusted p value to identify general trends for (A) NS5LGS, (B) NS5GTR, and (C) NS52xNLS compared to NS5WT. Genes with significant increases (log2 fold change > 0.5, padj <0.05) for NS52xNLS were used for heatmap analysis in Fig 6C. (TIF) [file ppat.1010100.s007.tif]

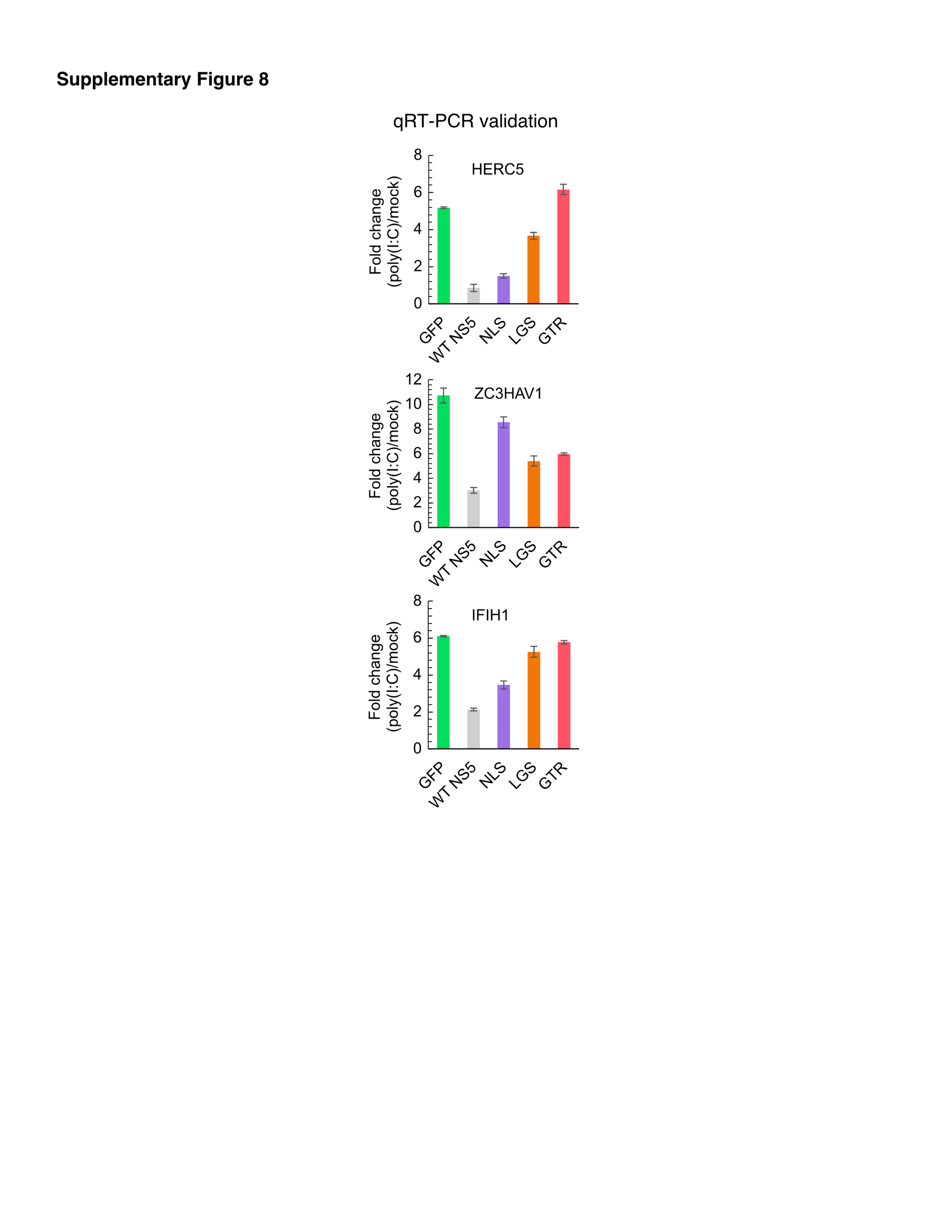

Supplement: S8 Fig — qRT-PCR was performed on PAF1-dependent immune response genes from Fig 7A. Fold changes were calculated using the ΔΔCt method and normalized to GAPDH as the house-keeping gene. GFP transfection was used as a positive control for poly(I:C) induction. (TIF) [file ppat.1010100.s008.tif]
