# Supplementary material for: Nuclear dengue virus NS5 antagonizes expression of PAF1-dependent immune response genes
Source: PLoS Pathog. 2021 Nov 19;17(11):e1010100. doi: 10.1371/journal.ppat.1010100 (PMC8641875; doi:10.1371/journal.ppat.1010100)
Supplement: Attachment — Submitted filename: Response to Reviewers.pdf [file ppat.1010100.s016.pdf]

Response to Reviewers

Part II – Major Issues: Key Experiments Required for Acceptance

Reviewer #1: - Fig 5A indicates that many flavivirus NS5 proteins interact with PAF1, but the immunoblots show an incredible multitude of bands of differing molecular weights. Could the authors provide some insight as to how they be sure of specificity if there are some many bands detected by the antibody? In all of the other assays shown which species is interacting with PAF1 directly then? Can this membrane be reprobed with anti-NS5 antibodies?

We used an anti-NS5 antibody to probe the whole cell lysate of cells expressing DENV NS5 with a C-terminal 2xStrep II tag. As seen in an updated supplementary figure (S4B Fig), the band pattern produced by the anti-NS5 antibody is almost identical to that produced by the anti-Strep antibody. This suggests that the bands we identify are N-terminal degradation products of full-length NS5 and retain the C-terminal 2xStrep II tag for our anti-Strep immunoblot. We have indicated this in the revised manuscript text, (lines 208-211).

Are the authors able to determine if there has been any genome methylation induced by presence or absence of PAF1? This may be important in determining the transcription regulation and ultimate mechanism

We probed whole cell lysate of parental A549, PAF1 rescue and PAF1 knockout cells with antibodies against histone methylation markers associated with PAF1C activity (H3K9me3, H3K27me3, H3K4me3, and H3K79me3). We did not observe any clear differences in global histone methylation. These data are added as a supplementary figure (S2 Fig) and described in the manuscript (lines 158-162). An assay like ChIP-qPCR or ChIP-seq, which would provide higher-resolution data of individual genes, could identify differences for specific PAF1-dependent genes, but is outside the scope of this manuscript. We thank the reviewer for this future direction.

Reviewer #2: 1. The viral replication experiment in Figure 1 lacks appropriate control cell lines. First, there is no wild-type equivalent cell line included for comparison. Because lentiviral transduction and clonal selection can alter cell responses, it is understandable that the authors did not compare their PFU data to a parental A549 cell. However, they need to compare their PAF1 KO cell line to a transduced/Cas9-ed cell in which PAF1 has not been manipulated. A scramble or untargeted gRNA control would work. A similar control is needed for the rescue cells (e.g. expression of a control protein like GFP in the KO background). It is definitely encouraging that the PAF1 and STAT2 rescues act similarly to WT A549s following polyI:C treatment (by virtue of clustering nicely in the Figure 2B PCA) but it is still important to show that the rescue phenotype is not being influenced by double transduction/selection and/or overexpression of an exogenous protein in the context of dengue virus infection.

Also relevant to Figure 1, there’s a question of how PAF1C protein expression in the rescue cell line compares to that in a WT A549 cell line. Is it possible to show a western blot comparing the PAF1C rescues to WT A549s (or better yet, the control cell lines requested above?)

The reviewer is correct in our original logical that we did not include parental A549 cells or non-targeting gRNA cells as a control since these cells did not undergo clonal selection like the PAF1 knockout cells did. To account for the two rounds of lentiviral transduction that the PAF1 rescue cells experienced, we created PAF1 knockout cells transduced with a GFP-expressing

lentivirus. We also created a non-targeting gRNA from parental A549 cells, though this cell line is not clonally selected like the PAF1 knockout cells. In new experiments included in a supplementary figure (S1C Fig), we show that the double transduction does not affect the overall behavior of PAF1 rescue cells compared to PAF1 knockout cells, and our conclusions regarding the ability of PAF1 to restrict DENV replication remain unchanged. It should be noted that the overall trends are the same, though the numerical titers are different because a new co-author performed these experiments with a different virus stock. The cell line with non-targeting gRNA does display different replication kinetics compared to the PAF1 rescue cells (S1D Fig), likely due to clonal selection that occurred in the PAF1 rescue cells to create the original knockout. An immunoblot comparing PAF1 expression in the cell lysate of the PAF1 rescue cells and non-targeting gRNA cell line shows lower expression in the PAF1 rescue cells (S1A Fig). This new data is included in the results (lines 106-118).

Given the differences in replication between the parental (bulk) control, and the PAF1 rescue (derived from a clonal knockout), we also addressed our use of the parental line as a comparison for RNA-seq analysis. First, it allows us to compare gene expression between the different knockouts (PAF1 and STAT2) using the same control. Second, the strong clustering of the parental and rescue cells in terms of gene expression behavior suggests that the differences in replication kinetics are driven by genes not captured in our gene expression analysis, and perhaps by another mechanism like changes in metabolism, survival, or cell health that is not apparent until the cells are stressed (*e.g.* by infection). This is addressed in the results (lines 137-142).

2. The real “slam dunk” experiment that’s missing here would be to show that reintroducing PAF1-interaction mutant versions of NS5 into DENV2 impacts the expression of PAF1-dependent genes in the context of infection. While polyI:C is a reasonable proxy for DENV2-driven innate immune activation, the dynamics and levels of innate immune gene induction in a polyI:C transfection are vastly different from that of a bone fide viral infection. Likewise, NS5 levels are surely different in an overexpression cell line vs. a DENV2-infected cell. A set of parallel experiments to those in Figure 6, but with WT DENV2 and mutant DENV2, would significantly strengthen the manuscript’s conclusions.

We agree with the reviewer that creating DENV2 infectious mutants would be the ideal way to test the importance of the NS5-PAF1C interaction during infection. Unfortunately, generating a viable mutant of DENV2 was not achievable within our limited time. We would first need to identify single mutants that disrupt the interaction, since a triple mutation has a high likelihood of being lethal. Moreover, even if single mutations were identified, these mutations could affect NS5 RdRP or MTase activity. We would need to perform careful *in vitro* biochemical assays to show that these enzyme activities were not affected to rigorously attribute any defects in replication to innate immune antagonism. We feel that this is the subject of an entire manuscript on its own, and would crowd out the valuable information gleaned here on PAF1- and STAT2-dependent gene expression, the biochemical determinants of the NS5-PAF1C interaction, and NS5 antagonism of innate immunity using our reductionist ectopic expression approach. We look forward to pursuing this as a future direction.

Reviewer #3: Separation of the STAT2 and PAF1 binding domains of NS5 is critical for the interpretation of the data in the manuscript. In Fig 5D, STAT2 binding to the NS5 mutants is dramatically reduced (but still present). While binding of all PAF1C components to NS5 looks to be completely abrogated in the same experiment, the levels of binding of wild-type NS5 to PAF1C components are also lower than for STAT2. The distinction between abrogated PAF1C binding and reduced (but still present) STAT2 binding may therefore simply result from

exposure differences in the western blot. It would be helpful to see a longer exposure of the PAF1C blots to show more convincingly that binding of PAF1C components are indeed completely abrogated. Quantification of binding levels would also be helpful in this regard.

We could not reprobe the previous blots, so we repeated the experiment in Fig 5D. We limited our immunoblot analysis to Strep (for NS5), PAF1 and GAPDH (as a negative control) for this additional experiment since loss of PAF1 interaction is a good indicator for loss of interaction with the rest of the complex. Expression of NS5 mutants was not even, so we adjusted loading of Strep-AP samples to have near equal amounts of WT and mutant NS5. Even with a long exposure (5 times longer than short exposure for each antibody), we detected no PAF1 binding, suggesting that these mutations truly break the NS5-PAF1C interaction. We tried even longer exposures, but these resulted in too much background signal for each antibody. We include these results in a supplementary figure (S5B Fig). We did not quantify this because there was no PAF1 signal to quantify for the mutant affinity purifications. Data are added to the results (lines 252-253).

Part III – Minor Issues: Editorial and Data Presentation Modifications
Please use this section for editorial suggestions as well as relatively minor modifications of existing data that would enhance clarity.

Reviewer #1: - the citation list is focused primarily on Dengue reports but perhaps could be extended to other reports where RNA-seq has been used to investigate flavivirus infection and NS5 expression, particularly as the authors are indicating a potential conserved role here

Flavivirus infection includes many different inputs that could impact gene expression, so we focused our discussion on NS5 gene expression studies. We have expanded the discussion to include more nuances related to West Nile virus RNA-seq datasets, and more limited analysis of gene expression for Zika virus, and duck Tembusu virus (lines 370-383). Importantly, this discussion highlights the potential of performing systematic comparative studies across flavivirus NS5 proteins under similar conditions (*e.g.* cell type, immune stimulation, timing, and data analysis).

Reviewer #2: Minor comments:

Line 48: unclear what methyltransferase activity is being referenced here.
We have clarified this sentence to refer to both the N7 and 2'-O methyltransferase activity (line 49).

Line 91: the meaning of this sentence is ambiguous. Is "the measurement of antigen-positive cells" the way in which you experimentally showed that PAF1C restricts DENV2 replication or is that the mechanism through which PAF1C restricts DENV2 replication?

We clarified this sentence to indicate that "measurement of DENV antigen-positive cells" was the experimental technique used to measure DENV replication (lines 98-99).

PAF1C's role in controlling RNAPII pausing and transcriptional elongation has been studied extensively but there is very little mention of it here. Could the authors elaborate in the discussion on the mechanistic role of PAF1C in the innate immune response and speculate as

to why certain genes like HERC5, TRAF2, CGAS, etc. would be sensitive to control by PAF1C?

In a new analysis leveraging published data on RNA Pol II pausing (ref 58), we explored the pausing index of PAF1-dependent genes, dividing them into anti-DENV and pro-DENV groups. We found anti-DENV PAF1-dependent genes were significantly more paused than pro-DENV PAF1-dependent genes. These data are presented in a new supplementary table (S7 Table). This suggested that NS5 antagonizes the positive regulatory role on RNA Pol II release for this subset of genes. We have added discussion on these potential mechanisms for PAF1-dependent regulation of specific immune response genes (lines 385-404).

Figure 4 figure legend is confusing because the MTase domain itself is at the N-terminus of the protein. Perhaps “A C-terminal portion of the NS5 MTase domain is responsible for interaction with PAF1”

We have incorporated this suggestion into the legend for Fig 4.

Additional detail is needed in the Results and/or methods sections as to how PAF1 KO and rescue cell line selections were carried out (e.g. Were the rescue cells made from clonal KOs? Did the rescue cells undergo multiple rounds of cloning?). If the rescues are not clonal, could we see an IF image showing how uniform expression of PAF1 is across multiple cells in the population?

The PAF1 knockout cells were made by clonal selection. The PAF1 rescue cells were made from the clonal PAF1 knockout cells, but were not subjected to an additional round of clonal selection. We have clarified this in the text (lines 434-436). We also provide new data characterizing the heterogeneity of PAF1 expression in the parental and PAF1 rescue line. Using immunofluorescence microscopy, we find that variation in PAF1 expression across the cell population is similar in PAF1 rescue cells compared to parental A549 cells. Immunofluorescence intensity differs from the immunoblot data, possibly because different antibodies were used for these assays. These data are included in a new supplementary figure (S1E-F Fig) and described in the text (lines 118-122).

Could the authors comment on the nature of the lower molecular weight bands in the anti-Strep blots in Figure 5A and 5B?

This is similar to a comment from Reviewer 1. We probed a whole cell lysate of NS5-transfected cells to show that most of these lower molecular weight bands bind anti-NS5 antibody. Thus the are likely N-terminal degradation/cleavage products of full length NS5. We include this data in supplementary figure (S4B Fig) along with text to describe the finding (line 208-211).

If I’m understanding this correctly, Figure 6 experiments look at parental A549 cells that have been transfected with different NS5 mutants. Are these proteins tagged? Can they be detected by western blot (against the tag, if they are tagged, or with an anti-NS5 antibody if they are not). Differences in expression of the proteins could impact the ability of these proteins to regulate gene expression in important ways and needs to be controlled for.

We used an anti-Strep antibody to probe the whole cell lysate of A549 cells transfected with the different NS5 constructs. All constructs show similar levels of expression. Consequently, differences in host gene expression are not likely due to different expression levels of NS5

mutants. These data are included in a supplementary figure (S6A Fig) and described in the text (lines 261-263).

Language in line 227 is confusing. What knockout cells are you referring to?

This was a typo since the comparison is with parental A549 cells. We have clarified this in the text (line 265).

Reviewer #3: 1. What are the levels of PAF1 in wild-type AF549 cells (Fig 1)? The authors only show PAF1 levels in PAF1 rescue cells, but the STAT2 rescue cells have lower STAT2 levels compared to wild-type AF549 cells (Fig S1). Gene expression and DENV replication is only shown in PAF1 knock-out cells relative to rescue cells, so it would be important to know how PAF1 expression compares in wild-type and rescue cells.

We include an immunoblot showing expression levels of PAF1 rescue cells and non-targeting gRNA cells. Similar to the STAT2 expression in the STAT2 rescue cells, PAF1 expression is lower in the PAF1 rescue cells. We include these data in a supplementary figure (S1A Fig) and describe the results in the text (lines 116-118). Please note that we also performed immunofluorescence microscopy on PAF1 in the rescue and parental cells (S1E-F Fig), as requested by Review 2 (lines 118-122). Please see our comments on these results above.

2. A loading control should be shown for the STAT2 western blot in Fig S1.

We have corrected this oversight.

3. When the authors refer to the nuclear localisation of NS5 (throughout the manuscript), it could be clarified that this refers to steady-state nuclear localisation. NS5 from different DENV serotypes/strains can appear more or less nuclear at steady state during infection, but likely all NS5 shuttles in and out of the nucleus albeit with a different steady state equilibrium. This could also be introduced better in the paragraph starting on line 56.

The paragraph describing nuclear localization of NS5 has been updated to highlight that we are considering steady-state nuclear localization of different serotypes, and the DENV serotype 2 16681 strain that we used (lines 57-63, 67 and 69).

4. The LGS/GTR mutants could be better explained in the legend to Fig 5.

We updated the legend to Fig 5 to better describe these mutations.
